# Sex differences in brain atrophy in multiple sclerosis

**DOI:** 10.1186/s13293-020-00326-3

**Published:** 2020-08-28

**Authors:** Rhonda R. Voskuhl, Kevin Patel, Friedemann Paul, Stefan M. Gold, Michael Scheel, Joseph Kuchling, Graham Cooper, Susanna Asseyer, Claudia Chien, Alexander U. Brandt, Cassandra Eve Meyer, Allan MacKenzie-Graham

**Affiliations:** 1 Department of Neurology, University of California, Los Angeles, 635 Charles E. Young Drive South, Gordon Neuroscience Research Building, Los Angeles, CA 90095 USA; 2 Experimental and Clinical Research Center, Max Delbrueck Center for Molecular Medicine and Charité – Universitätsmedizin Berlin, Corporate member of Freie Universität Berlin, Humboldt-Universität zu Berlin, and Berlin Institute of Health, Berlin, Germany; 3 NeuroCure Clinical Research Center, Charité – Universitätsmedizin Berlin, Corporate member of Freie Universität Berlin, Humboldt-Universität zu Berlin, and Berlin Institute of Health, Berlin, Germany; 4 Institute for Neuroimmunology and Multiple Sclerosis (INIMS), Center for Molecular Neurobiology, Universitätsklinikum Hamburg-Eppendorf, Hamburg, Germany; 5 Department of Psychiatry and Medical Department, Division of Psychosomatic Medicine, Campus Benjamin Franklin, Charité – Universitätsmedizin Berlin, Corporate member of Freie Universität Berlin, Humboldt-Universität zu Berlin, and Berlin Institute of Health, Berlin, Germany; 6 Institute of Neuroradiology, Charité – Universitätsmedizin Berlin, Corporate member of Freie Universität Berlin, Humboldt-Universität zu Berlin, and Berlin Institute of Health, Berlin, Germany; 7 Departments of Neurology and Neuropsychiatry, Charité – Universitätsmedizin Berlin, Corporate member of Freie Universität Berlin, Humboldt-Universität zu Berlin, and Berlin Institute of Health, Berlin, Germany; 8 Einstein Center for Neurosciences, Berlin, Germany; 9 Department for Psychiatry and Psychotherapy, Charité – Universitätsmedizin Berlin, Corporate member of Freie Universität Berlin, Humboldt-Universität zu Berlin, and Berlin Institute of Health, Berlin, Germany

**Keywords:** Multiple sclerosis, Sex differences, Neuroimaging, Neurodegeneration, Disability progression

## Abstract

**Background:**

Women are more susceptible to multiple sclerosis (MS) than men by a ratio of approximately 3:1. However, being male is a risk factor for worse disability progression. Inflammatory genes have been linked to susceptibility, while neurodegeneration underlies disability progression. Thus, there appears to be a differential effect of sex on inflammation versus neurodegeneration. Further, gray matter (GM) atrophy is not uniform across the brain in MS, but instead shows regional variation. Here, we study sex differences in neurodegeneration by comparing regional GM atrophy in a cohort of men and women with MS versus their respective age- and sex-matched healthy controls.

**Methods:**

Voxel-based morphometry (VBM), deep GM substructure volumetry, and cortical thinning were used to examine regional GM atrophy.

**Results:**

VBM analysis showed deep GM atrophy in the thalamic area in both men and women with MS, whereas men had additional atrophy in the putamen as well as in localized cortical regions. Volumetry confirmed deep GM loss, while localized cortical thinning confirmed GM loss in the cerebral cortex. Further, MS males exhibited worse performance on the 9-hole peg test (9HPT) than MS females. We observed a strong correlation between thalamic volume and 9HPT performance in MS males, but not in MS females.

**Conclusion:**

More regional GM atrophy was observed in men with MS than women with MS, consistent with previous observations that male sex is a risk factor for worse disease progression.

## Introduction

Females are more susceptible to multiple sclerosis (MS) than males, with a ratio of approximately 3:1 [1–3]. Increased susceptibility of females occurs in many autoimmune diseases, suggestive of a fundamental sex-dependent immune mechanism as its etiology [4, 5]. Women have more robust immune responses to self and foreign antigens as compared to men, and this sex difference is observed across species [6, 7]. Sex differences can be due to sex hormones, sex chromosomes, or both [5]. Sex hormone effects on the immune response in MS and its animal models have been widely documented in the literature [8–10]. A sex chromosome effect on immune responses has been observed in MS models, where the XX genotype conferred a more pro-inflammatory response than the XY genotype [11, 12]. Finally, the female to male ratio in MS has increased in the past decades [1], likely due to gene-environment interactions [5].

Consistent with the importance of sex as a biological variable [13], there are sex differences not only in the immune system but also in the brain [14]. Healthy male brains are known to be significantly larger than those of females, and there are regional differences in substructure volumes that persist, even when accounting for differences in brain size [15–18]. Sex differences in the brain are observed across species from humans to mice [19–21].

The effect of sex appears to differ in the immune system versus the central nervous system (CNS) in MS [4, 5], since women are more susceptible to disease, but men are at higher risk for worse disability [22–25]. In a very large natural history study in relapsing-remitting MS (RRMS), men also demonstrated a shorter time to conversion to secondary progressive MS (SPMS) [26]. Together, these studies suggest the possibility that MS men may have a more severe neurodegenerative response to inflammatory attacks [5].

Brain atrophy, specifically gray matter (GM) atrophy, serves as a putative surrogate for neurodegeneration in MS. Regional differences in GM atrophy have been shown in MS, and clinical disabilities have been shown to correlate with GM atrophy in clinically eloquent neuroanatomic regions [27–31]. Regional differences in gene expression in astrocytes, microglia, and oligodendrocytes have also been shown in both health and disease [32–35], including a sex-specific astrocytic response to injury in an MS model [36]. Given the regional heterogeneity of the brain, we hypothesized that being female or male may show regional differences in GM atrophy in the brain during MS.

Here, sex differences in regional GM atrophy in MS were evaluated using a cohort of female and male MS subjects with age- and sex-matched healthy controls to reveal insights relevant to sex differences in neurodegeneration.

## Methods

### Patients

For this retrospective cross-sectional study, we screened data from 114 MS patients participating between July 2014 and August 2018 in an ongoing observational study at the NeuroCure Clinical Research Center at the Charité – Universitätsmedizin Berlin (VIMS study; EA1/163/12). Inclusion criteria were a minimum age of 18 years and the fulfillment of the 2010 McDonald criteria for MS [37]. Patients were excluded if they had a history of neurological diseases (other than MS), primary progressive MS, or were missing brain MRI data. We included 89 MS patients (RRMS: 79, SPMS: 10) in the study. We included 45 age- and sex-matched healthy controls (HCs) who had an imaging assessment from our imaging research database. Altogether, 134 subjects were included in this study, with 89 MS (52 females and 37 males) and 45 healthy controls (28 females and 17 males). Female MS subtype was 87% (45) RRMS, 10% (5) SPMS, and data was unavailable for 4% (2), whereas male MS subtype was 81% (30) RRMS, 14% (5) SPMS, and data was unavailable for 5% (2). Within female MS patients, 27% (14) were not on any disease-modifying treatment (DMT), 71% (37) were on a DMT (8 dimethyl fumarate, 7 fingolimod, 9 glatiramer acetate, 11 interferon beta, 2 teriflunomide), and data was unavailable for 2% (1). Within male MS patients, 22% (8) were not on any DMT, 70% (26) were on a DMT (3 dimethyl fumarate, 8 fingolimod, 3 glatiramer acetate, 3 interferon beta, 1 natalizumab, 8 teriflunomide), and data was unavailable for 8% (3). Regarding the potential contribution of comorbidities, only 1 patient was obese, 1 was a smoker, and none were diabetic. Hypertension was observed in 11 MS patients (6 female and 5 male) and 1 healthy control (male). All participants provided written informed consent prior to their inclusion in the study. The study was approved by the local ethics committee and was performed in accordance with the Declaration of Helsinki in its currently applicable version.

### Clinical testing

Patients were examined under supervision of a board-certified neurologist at the NeuroCure Clinical Research Center, Charité – Universitätsmedizin Berlin to obtain the Expanded Disability Status Scale (EDSS) score [38]. Standardized walking and upper extremity function were assessed using two trials of the timed 25-foot walk (T25FW) and two trials of the 9-hole peg test (9HPT) per hand, respectively. To test cognitive processing speed, the Symbol Digit Modalities Test (SDMT) was completed according to manual protocol (Smith, A., SDMT manual, Los Angeles, CA, USA: Western Psychological Services) using the oral form (as opposed to the written form) to eliminate the impact of fine or gross motor impairments on SDMT performance.

### MRI acquisition and image processing

All MRI data were acquired on the same 3T scanner (MAGNETOM Tim Trio Siemens, Erlangen, Germany) using a 3D high-resolution T1-weighted magnetization prepared rapid acquisition gradient echo (MPRAGE) sequence (TR/TE/TI = 1900/3.03/900 ms, FOV = 240 × 240 mm^2^, matrix 240 × 240, 176 slices, 1 × 1 × 1 mm^3^ resolution), as well as a 3D high-resolution fluid-attenuated inversion recovery sequence (FLAIR) (TR/TE/TI = 6000/388/2100 ms; FOV = 256 × 256 mm^2^, 1 × 1 × 1 mm^3^ resolution). T2-hyperintense white matter lesion segmentation for total lesion volume was semi-automatically performed on 3D FLAIR images of all subjects using the MATLAB SPM12 Lesion Segmentation Toolbox (LST) [39] lesion growth algorithm and manually checked and edited by two expert raters under the supervision of a board-certified radiologist (M.S.) using ITK-SNAP [40, 41]. Raters were blinded to sex, but not MS status.

Lesion in-painted MPRAGE images were processed in MATLAB (The MathWorks, Natick, MA) and examined with SPM12 [42] and the computational anatomy toolbox (CAT-12) [43], using an approach similar to that described previously [31]. In brief, the in-painted images were tissue classified into gray matter (GM), white matter (WM), and cerebrospinal fluid (CSF) and registered to MNI space using linear and non-linear transformations. The GM and WM segments were modulated for non-linear components of the transformation. This resulted in voxel-wise comparability between the subjects while correcting for differences in whole brain size. The GM segments were then smoothed with a Gaussian kernel (8 mm FWHM) for VBM. The statistical parametric maps generated by VBM are the result of statistical analyses conducted across the entire brain at a voxelwise level comparing all the female MS patients to all the female healthy controls and all the male MS patients to all the male healthy controls. All analyses were covaried for age and intracranial volume and the statistical parametric maps were corrected for voxel-wise multiple comparisons by controlling the false discovery rate (FDR) [44] at *p* = 0.05. GM, WM, and brain parenchymal fraction (BPF = GM + WM) volumes were computed as the sum of all voxel-wise volumes within spatially normalized and modulated GM and WM segments.

Subcortical structure volumes (thalamus and putamen) were quantified using the FreeSurfer software package version 6.0.1 segmentation pipeline [45, 46]. Cortical surface reconstruction was performed using a semi-automated approach [47]. To assess sex-specific cortical thickness differences between patients with MS and HCs, we then performed male and female vertex-wise analyses using a general linear model controlling for the effect of age. Two independent analyses compared thickness in (1) MS females vs. healthy females, and (2) MS males vs. healthy males. To control for multiple comparisons, we employed a cluster-wise correction using a Monte Carlo simulation with a *p* value set at < 0.05 [48].

### Statistical analysis

We summarized subject characteristics using descriptive statistics and compared both male and female MS patients and HCs using unpaired, heteroscedastic two-tailed *t* tests. Heteroscedastic tests were performed to account for differences in the variability of the measured variables between MS patients and healthy controls. Chi-squared test was used to test for differences in disease modifying treatment and MS subtype within the different sexes. FreeSurfer subcortical volumes, GM, WM, and BPF volumes between female HCs and female MS patients and between male HCs and male MS patients were compared using unpaired, heteroscedastic two-tailed *t* tests corrected for intercranial volume. The statistician was blinded to sex, but not MS status. *p* values for imaging analyses were adjusted for multiple comparisons by controlling for the false discovery rate (FDR) [49]. Effect sizes were calculated as Hedge’s *g*, interpreted as *g* > 0.20 (small effect); *g* > 0.50 (medium effect); and *g* > 0.80 (large effect). 9HPT, T25FW, and SDMT between female MS patients and male MS patients were also compared using unpaired, heteroscedastic two-tailed *t* tests. Pearson’s correlations (corrected for intracranial volume) between 9HPT and deep gray structure volumes were found by conducting linear regression analyses in R [50]. *p* values for clinical evaluations and correlations were adjusted for multiple comparisons by using the Bonferroni correction.

## Results

### Patient descriptive characteristics

A total of 89 MS (52 females and 37 males) and 45 healthy control (28 females and 17 males) brain MRIs were analyzed. There were no differences between MS female and MS male subjects (Table 1) in mean age, duration of disease since diagnosis, or composite disability scores, as measured by the EDSS. There were also no differences in T2-hyperintense (FLAIR) white matter lesion counts or volumes and no differences in the proportion on disease modifying treatments or MS subtype (see methods). There was also no difference in age between MS females versus healthy females and MS males versus healthy males.

**Table 1:** Descriptive characteristics

Descriptive characteristics	Female MS patients (*n* = 52)	Male MS patients (*n* = 37)	*p* value	Female healthy controls (*n* = 28)	Male healthy controls (*n* = 17)	*p* value
Age						
Mean ± SD (years)	42.1 ± 12.4	40.7 ± 11.7	0.60	38.2 ± 12.5	37.2 ± 15.7	0.83
Median, IQR	42.3, 31.8–50.7	40.0, 32.0–49.7		35.3, 29.7–44.0	30.5, 26.1–40.6	
Range	18.1-66.8	20.3-64.1		20.5–69.0	21.5–68.3	
Duration of MS (from Dx)						
Mean ± SD (years)	8.5 ± 7.7	8.5 ± 6.8	1.00	n/a	n/a	n/a
Median, IQR	5.9, 1.9–14.5	7.3, 2.2–13.0		n/a	n/a	
Range	0.3-28.6	0.0-23.9		n/a	n/a	
EDSS						
Mean ± SD	2.4 ± 1.4	2.3 ± 1.5	1.00	n/a	n/a	n/a
Median, IQR	2.0, 1.5–3.0	2.0, 1.0–3.0		n/a	n/a	
Range	0–6.5	0–6.0		n/a	n/a	
T2-hyperintense (FLAIR) white matter lesion count						
Mean ± SD	33.9 ± 22.7	35.1 ± 25.4	0.83	4.4 ± 7.1	6.9 ± 12.0	0.43
Median, IQR	35, 14.8–48	30, 17-48		2, 0–4.3	1, 0–6	
Range	2–107	0-128		0–30	0–43	
T2-hyperintense (FLAIR) white matter lesion volume						
Mean ± SD (cc)	8.1 ± 10.8	8.1 ± 8.2	0.98	0.3 ± 0.5	1.0 ± 3.5	0.39
Median, IQR	4.8, 1.2–10.5	5.7, 1.9–11.4		0.1, 0.0–0.2	0.0, 0.0–0.2	
Range	0.1–50.0	0.0–31.8		0.0–2.0	0.0–14.6	

Descriptive characteristics of the MS and healthy control populations

### Voxel-based morphometry to detect regional GM atrophy in MS females and MS males

**Fig. 1:**
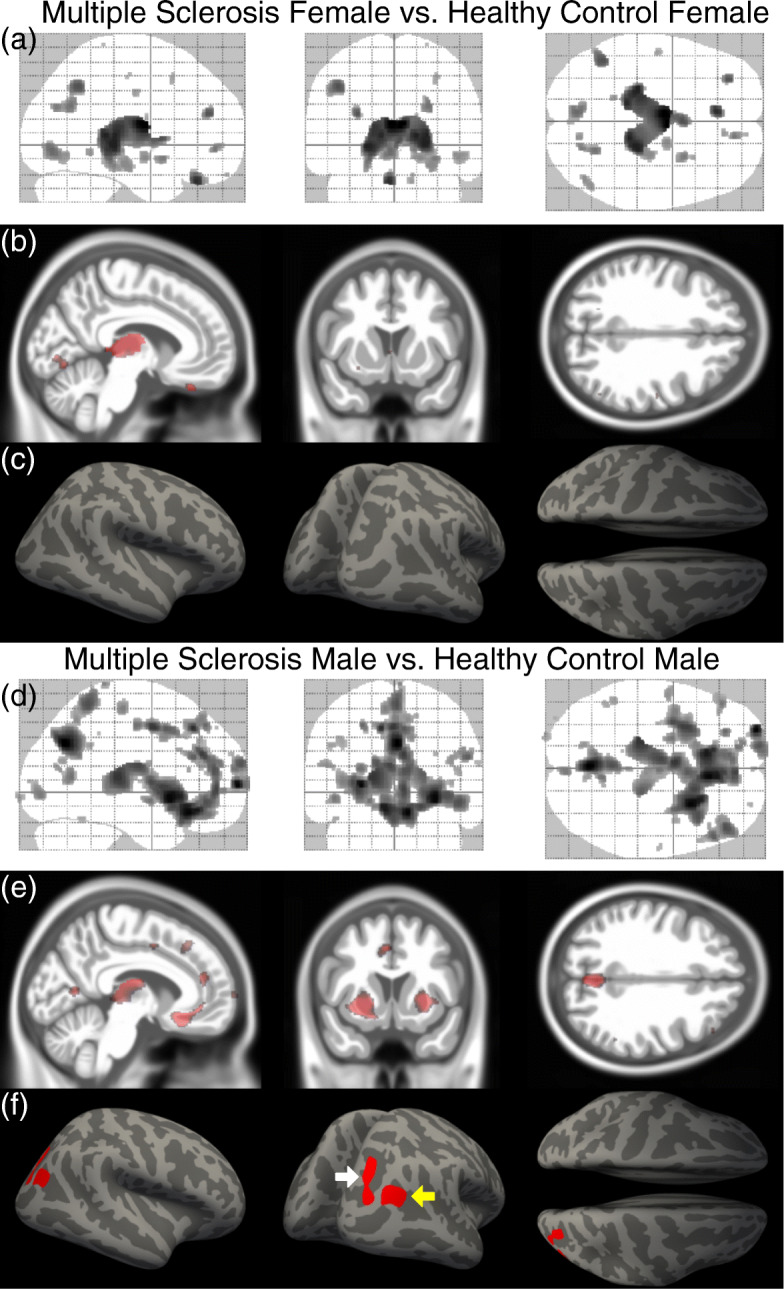
Gray matter atrophy and cortical thinning in female and male multiple sclerosis patients compared to healthy controls. **a** Maximum intensity projections of the statistical parametric map of GM loss in all female MS patients compared to all female healthy controls (*p* < 0.05, FDR corrected) overlaid on the SPM glass brain demonstrating significance clusters in the thalamus. **b** Sagittal, coronal, and axial sections through the statistical parametric map of GM loss highlighting atrophy in the thalamus (sagittal view) overlaid on the CAT12 mean IXI template. **c** FreeSurfer cortical thinning maps demonstrating no statistically-significant cortical thinning in female MS patients compared to healthy controls. **d** Maximum intensity projections of the statistical parametric map of GM loss in all male MS patients compared to all male healthy controls (*p* < 0.05, FDR corrected) overlaid on the SPM glass brain demonstrating significance clusters in the thalamus, putamen, the precuneus, and medial frontal cortex. **e** Sagittal, coronal, and axial sections the statistical parametric map of GM loss highlighting atrophy in the thalamus (sagittal view), putamen (coronal view), and precuneus (axial view) overlaid on the CAT12 mean IXI template. **c** FreeSurfer cortical thinning maps demonstrating statistically-significant cortical thinning in the inferior parietal lobule (yellow arrow) and the superior and transverse occipital sulci (white arrow) in male MS patients compared to healthy controls

A biology-driven approach, namely voxel-based morphometry, showed deep GM loss in the thalamic region in MS females (Fig. 1a, b) and in MS males (Fig. 1d,e), each as compared to their respective age- and sex-matched HCs. In addition, MS males showed significance clusters of GM loss in the putamen, precuneus, and medial frontal cortex (Fig. 1d ,e), which were more pronounced than that in MS females (Fig. 1a, b).

### Quantification of atrophy of deep GM substructures in MS females and MS males

Based on the voxel-based morphometry findings (Fig. 1), deep GM substructure volumes were determined using FreeSurfer (Table 2). Consistent with voxel-based morphometry data, decreased thalamic volume was observed in MS females and MS males as compared to their respective healthy age- and sex-matched controls. This finding is consistent with previous observations of thalamic atrophy occurring early in MS [51–54]. Notably, MS males had more atrophy in the thalamus and putamen than MS females, each as compared to their respective HCs.

**Table 2:** MRI measures.

MRI measures	Healthy controls Mean ± SD cc (*n*)	Multiple sclerosis Mean ± SD cc (*n*)	Absolute difference cc	Percent difference	^‡^*p* value *q* value Hedge’s *g*
FreeSurfer thalamus					
Female	14.88 ± 1.10 (28)	13.91 ± 1.62 (52)	− 0.98	− 6.8%	*p* = 0.0021 *q* = 0.0070 *g* = 0.66
Male	16.67 ± 0.98 (16)	15.24 ± 1.47 (37)	− 1.43	− 9.0%	*p* = 0.00015 *q* = 0.00075 *g* = 1.06
FreeSurfer putamen					
Female	9.50 ± 0.97 (28)	8.86 ± 1.17 (52)	− 0.65	− 7.1%	*p* = 0.010 *q* = 0.020 *g* = 0.58
Male	11.44 ± 0.88 (16)	10.14 ± 1.26 (37)	− 1.30	− 12.0%	*p* = 0.00011 *q* = 0.00075 *g* = 1.12
Brain parenchymal fraction					
Female	1088.3 ± 93.9 (28)	1029.9 ± 85.8 (52)	− 58.4	− 5.5%	*p* = 0.0086 *q* = 0.020 *g* = 0.66
Male	1235.8 ± 89.0 (17)	1180.8 ± 78.3 (37)	− 55.0	-4.5%	*p* = 0.037 *q* = 0.041 *g* = 0.67
Total gray matter					
Female	585.6 ± 51.1 (28)	555.6 ± 48.6 (52)	− 30.0	− 5.2%	*p* = 0.0139 *q* = 0.023 *g* = 0.61
Male	658.0 ± 48.8 (17)	624.1 ± 46.6 (37)	− 33.9	− 5.3%	*p* = 0.0226 *q* = 0.028 *g* = 0.72
Total white matter					
Female	502.7 ± 52.1 (28)	474.3 ± 46.0 (52)	− 28.4	− 5.8%	*p* = 0.019 *q* = 0.027 *g* = 0.59
Male	577.7 ± 53.7 (17)	556.7 ± 44.1 (37)	− 21.1	− 3.7%	*p* = 0.17 *q* = 0.17 *g* = 0.44

FreeSurfer subcortical structure volumes and voxel-based morphometry (VBM) gray matter (GM), white matter (WM), and brain parenchymal fraction (BPF) volumes from female and male MS patients and healthy controls. ‡ *p* is the uncorrected *p* value, *q* is the *p* value adjusted for multiple comparisons by controlling the FDR, and *g* is the Hedge’s *g* value.

### Cortical thinning in MS females and MS males

Regional cortical thinning was determined using FreeSurfer. Cortical thinning was observed in MS males, but not in MS females, each as compared to their respective HCs (Fig. 1c, f). The cortical thinning in MS males localized to the right intraparietal sulcus (IPS) (Fig. 1f, white arrow), a region known to be a core part of the dorsal attention network (frontoparietal connectivity between the frontal eye fields anteriorly and the intraparietal sulcus posteriorly) which is responsible for sustained, selective, executive attention [55, 56], as well as the middle occipital gyrus which is involved in visual spatial attention (Fig. 1f, yellow arrow) [55, 57].

### Gray matter, white matter, and brain parenchymal fraction volumes in MS females and MS males

GM, WM, and brain parenchymal fraction (BPF) volumes were measured in MS females and MS males, each as compared to their respective HCs (Table 2). GM volumes were each significantly decreased by approximately 5% in both sexes. WM volumes were significantly decreased in MS females, while a decrease in WM volume in MS males did not reach significance. BPF volumes were each significantly decreased in both MS females and MS males.

### Clinical disability scores in MS females and MS males

MS males had worse upper extremity function as shown by significantly worse performance on the 9HPT than MS females (Table 3). We did not observe a difference between MS male and MS female walking speeds, as assessed by the T25FW. There was also no difference in cognitive processing speed performance, as measured by the oral form of the SDMT.

**Table 3:** Clinical measures

Clinical measures	Female MS Mean ± SD (*n*)	Male MS Mean ± SD (*n*)	Absolute difference	Percent difference	^‡^*p* value *q* value Hedge’s *g*
9-Hole peg test	19.6 ± 3.0 s (46)	22.3 ± 5.4 s (30)	2.7 s	13.1%	*p* = 0.016 *q* = 0.048 *g* =0.66
Timed 25-foot walk	4.69 ± 1.0 s (45)	4.68 ± 1.7 s (27)	− 0.01 s	− 0.1%	*p* = 0.98 *q* = 1.00 *g* = 0.001
Symbol digit modalities test	59.6 ± 14.7 (44)	55.8 ± 14.3 (32)	− 3.8	− 6.5%	*p* = 0.20 *q* = 0.60 *g* = 0.26

9-hole peg test (9HPT), timed 25-foot walk (T25FW), and symbol-digit modalities test (SDMT) from female and male MS patients. ‡ *p* is the uncorrected *p* value, *q* is *p* value adjusted for multiple comparisons using the Bonferroni correction, and *g* is Hedge’s *g*

### Correlations between 9HPT and subcortical GM volumes

When MS female and male data were pooled, we observed a statistically significant inverse correlation between performance on the 9HPT and thalamic volume (Table 4). Since increased 9HPT times indicate worse performance, this result suggests that worse performance correlates with smaller thalami. Importantly, when examined separately in each sex, male MS patients exhibited a significant inverse correlation between performance on the 9HPT and thalamic volume, whereas female MS patients did not.

**Table 4:** Correlations

Correlations	‡All MS	Female MS	Male MS
Thalamus volume vs. 9HPT	*r* = − 0.43 *p* = 0.00020 *q* = 0.0012	*r* = − 0.29 *p* = 0.052 *q* = 0.31	*r* = − 0.58 *p* = 0.0025 *q* = 0.015
Putamen volume vs. 9HPT	*r* = − 0.35 *p* = 0.0026 *q* = 0.016	*r* = − 0.26 *p* = 0.083 *q* = 0.50	*r* = − 0.51 *p* = 0.0087 *q* = 0.052

Correlations between the 9-hole peg test (9HPT) and thalamus volume and between the 9HPT and putamen volume in female and male MS patients. ‡ *r* is Pearson’s *r*, *p* is the uncorrected *p* value, and *q* is *p* value adjusted for multiple comparisons using the Bonferroni correction

Similarly, when MS female and MS male data were pooled, we observed an inverse correlation between performance on the 9HPT and the putamen volume. Again, when examined in each sex, male MS patients exhibited a trend toward an inverse correlation between performance on the 9HPT and putamen volume, whereas female MS patients did not.

## Discussion

Here, we address an enigma regarding sex differences in MS. While women have more robust immune responses and are more susceptible to MS and other autoimmune diseases [6], being male is a risk factor for worse MS disability progression [5]. Since robust immune responses and relapses are thought to contribute to neurodegeneration and disability progression, one would expect females and not males, to demonstrate worse MS disability progression. However, since that is not the case, the effect of sex appears to differ based on inflammation versus neurodegeneration. We previously hypothesized that MS men may have a more severe neurodegenerative response to immune attack [5], and addressed this in a MS preclinical model. Bone marrow chimeras were used to separate effects of sex on the immune system versus the CNS. The XX genotype had increased pro-inflammatory immune responses [11], while the XY genotype had a more severe neurodegenerative response to a given immune attack [58].

Here, using independent and complementary approaches in clinical MS data, we found that MS men demonstrated worse localized GM atrophy than MS women, each as compared to their respective healthy age- and sex-matched controls. This difference in regional GM atrophy was not the result of MS males having older age, longer disease duration, or more comorbidities as compared to MS females. Since GM atrophy is a sensitive putative biomarker for clinical disability progression, these results provide insights into previous observations that male sex confers increased risk for disability progression [22–26]. Our results now warrant a longitudinal study of regional GM atrophy and disability progression rates over time in MS men and MS women clinically matched at baseline.

When examining sex differences in regional brain atrophy in cross-sectional data, there are two critical factors. First, one must include age- and sex-matched healthy controls. Given the known major sex differences in overall brain size and substructure volumes between healthy females and males for [15–18], each sex should be compared to it respective HC. This major confound of the sex difference in healthy brains is thereby removed by comparing MS female brains with healthy female brains and MS male brains with healthy male brains. This will reveal the effect of the MS disease process within each sex.

The second critical factor when examining sex differences in brain atrophy is to account for regional differences in the brain. Regional differences in gene expression in CNS cells have been shown in both health and disease [32–35], and regional differences in GM atrophy have been shown in MS [27–29]. The study of whole GM atrophy can dilute and leave out significant and potentially clinically eloquent regional GM atrophy resulting in less sensitivity in detection. Evaluation of whole cerebral cortical atrophy also pools together highly disparate cortical regions based on known anatomy, gene expression, and function. Since sex hormones and sex chromosomes would not act homogeneously across cells in the entire brain, differential effects on cells in specific CNS regions can be missed when examining whole GM or whole cerebral cortex. We previously hypothesized that sex differences in neurodegeneration in MS would be region-specific [33, 35, 36], and our data here on sex differences in GM atrophy are consistent with this hypothesis.

Aligned with a region-specific approach to sex differences in MS, a disability-specific approach is also warranted. Previous studies have suggested that cognitive disability in particular may be worse in males as compared to females with MS [25], and differences in atrophy of major GM structures have been correlated with cognitive disability [28, 54, 59–63]. Further studies are needed comparing MS women and MS men using biology-driven functional connectivity analysis [26, 35–67] and voxel-wise mapping of localized GM atrophy aligned with cognitive disability [30, 31]. Sex differences in other disabilities such as in walking or vision, for example, should also be mapped to contrast with cognitive disability maps. Given the known sex differences in the healthy brain [14–18], age- and sex-matched healthy controls for these regional analyses are also required. The importance of studying each sex independently in a disease has been widely recognized [13, 14]. Our observation that 9HPT performance was impaired in MS men, but not MS women, and that this clinical impairment correlated with more atrophy of the thalamus in MS men, but not MS women, underscores the importance of studying each sex independently. Thalamic atrophy in early MS has been demonstrated previously [51–54], although not in a sex-specific manner. With regard to cortical thinning, this was previously observed in the right superior and inferior parietal gyri of the Desikan-Killiany Atlas [68] in a pooled dataset where the female to male ratio was 1.6:1 [51]. Interestingly, there was substantial overlap with cortical thinning we observed in the right intraparietal sulcus of the Destrieux Atlas [55] in MS males (Fig. 1f).

A limitation of this study is the moderate sample sizes, therefore future studies with larger sample sizes focusing on correlations between clinical disabilities and regional atrophy are warranted in each sex. Also, all the data here are from a single cohort in Berlin, Germany. Whether these results can be extrapolated to patients from other MS cohorts warrants further investigation.

In MS, precision medicine requires targeting the most responsive population with the most appropriate anti-inflammatory treatment. In the future, neuroprotective treatments must be designed with precision using a sex-specific and CNS region-specific approach. This will advance the development of disability-specific neuroprotective treatments optimally tailored for each sex. Finally, sex differences in regional GM atrophy shown here in MS can serve as a roadmap for similar analyses in other neurodegenerative diseases that exhibit sex differences [14], such as Alzheimer’s disease [69, 70] and Parkinson’s disease [71, 72].

### Perspectives and significance

In summary, localized brain regions were found to undergo worse atrophy in multiple sclerosis men than in multiple sclerosis women, each as compared to their respective healthy, age- and sex-matched controls. These results underscore the importance of studying sex differences in each organ system, since MS women are more susceptible to disease which is thought to reflect differences in the immune system, but MS men have worse disability progression which is thought to reflect differences in the central nervous system. Also, the approach used here can be applied to other neurodegenerative diseases characterized by a sex difference. Specifically, mapping sex differences in brain regions during neurodegenerative diseases can serve as a foundation for future neuroprotective treatment trials targeting these regions in each sex.

## Data Availability

The datasets used and/or analyzed during the current study are available from the corresponding author on reasonable request.
